# Spiritual care practices in hospices in the Western cape, South Africa: the challenge of diversity

**DOI:** 10.1186/s12904-020-00704-z

**Published:** 2021-01-10

**Authors:** Ronita Mahilall, Leslie Swartz

**Affiliations:** 1 Department of Psychology, Stellenbosch University, Private Bag X4, Matieland, Stellenbosch, 7745 South Africa; 2 Department of Psychology, Stellenbosch University, Private Bag X1, Matieland, Stellenbosch, 7602 South Africa

**Keywords:** Spiritual care, South Africa, Diversity, Palliative care, Hospice

## Abstract

**Background:**

South Africa is a very diverse middle-income country, still deeply divided by the legacy of its colonial and apartheid past. As part of a larger study, this article explored the experiences and views of representatives of hospices in the Western Cape province of South Africa on the provision of appropriate spiritual care, given local issues and constraints.

**Methods:**

Two sets of focus group discussions, with 23 hospice participants, were conducted with 11 of the 12 Hospice Palliative Care Association registered hospices in the Western Cape, South Africa, to understand what spiritual care practices existed in their hospices against the backdrop of multifaceted diversities. The discussions were analysed using thematic analysis.

**Results:**

Two prominent themes emerged: the challenges of providing relevant spiritual care services in a religiously, culturally, linguistically and racially diverse setting, and the organisational context impacting such a spiritual care service. Participants agreed that spiritual care is an important service and that it plays a significant role within the inter-disciplinary team. Participants recognised the need for spiritual care training and skills development, alongside the financial costs of employing dedicated spiritual care workers. In spite of the diversities and resource constraints, the approach of individual hospices to providing spiritual care remained robust.

**Discussion:**

Given the diversities that are largely unique to South Africa, shaped essentially by past injustices, the hospices have to navigate considerable hurdles such as cultural differences, religious diversity, and language barriers to provide spiritual care services, within significant resource constraints.

**Conclusions:**

While each of the hospices have established spiritual care services to varying degrees, there was an expressed need for training in spiritual care to develop a baseline guide that was bespoke to the complexities of the South African context. Part of this training needs to focus on the complexity of providing culturally appropriate services.

## Background

Most of what is known about spiritual care comes from well-resourced countries in the global North [1–3]. This article deals with spiritual care in South Africa (SA), a middle-income country, and also a country which is linguistically, racially, culturally and spiritually diverse [4], with inequalities [5], and with a complex political past and present. Apartheid, a system of racial oppression which completely segregated and divided SA, formalising into law many of the informal aspects of segregation and oppression common in colonial contexts, had a profound effect on the emotional and spiritual lives of all its citizens, and despite SA having been a democracy since 1994, it remains a divided society with a long shadow of the past still affecting contemporary spiritual life, health, and, indeed, all aspects of society [6–10].

Spirituality can be defined as a “way individuals seek and express meaning and purpose and the way they experience their connectedness to the moment, to self, to others, to nature, and to the significant or sacred” [11]. Clearly, in thinking about spiritual care in Africa, and in SA specifically, both the historical marginality and the continuing vitality of local approaches to spirituality need to be borne in mind [12, 13].

The modern hospice movement began in the global North in the 1950s with Dame Cicely Saunders [14]. Hospices in Africa began in Zimbabwe in 1980, followed by SA in 1987 and Kenya in 1990. Most countries have palliative care associations that coordinate the work of hospices, especially with regard to sharing best practices, care standards and protocols [15]. The Hospice Palliative Care Association (HPCA) of SA is one such association. The population of SA was 58,78 million according to the 2019 mid-year population estimates [16]. The total number of deaths registered at the South African Department of Home Affairs and processed by Stats SA in 2017 were 446,544. Although it was found that most male deaths are related to tuberculosis and most female deaths are related to diabetes mellitus, cancers are still the leading cause of terminal diagnosis [17]. “The estimated need for palliative care, using only mortality data, is that 0.52% of the population require palliative care in any year” [15].

Arising out of a broader study on spiritual care in SA, this article explores how the hospices in the Western Cape Province of SA offer spiritual care services while navigating the rugged terrain of racial prejudices, social injustices, and cultural segregation.

## Theoretical framework

In addressing questions of cultural and spiritual diversity in palliative care, we are influenced by the research tradition on explanatory models of illness and disease, as first formulated by Kleinman and developed by a range of other authors [18–20]. For Kleinman and those that follow his work, all people interpret illness and misfortune in the light of cultural categories that are part of their social world – and both patients and those who care for them have elaborated cultural explanations of illness and distress. Given the socio-political context of SA, it is important to note that explanatory models and systems are profoundly affected by social factors, including histories of exclusion and oppression – culture never exists apart from the socio-political context [21–23].

## Methods

Ethical approval was obtained from Stellenbosch University (10237) as well as Hospice Palliative Care Association (HPCA) (02/19). HPCA is a national association operating in all nine provinces in SA and 51 health districts. Some of its voluntary hospices offer hospice palliative care, some home-based care, and others both services. We conducted two focus group discussions (FGDs) with employees of hospices in the Western Cape. Eleven of the 12 registered member organisations (hospices) in the province agreed to participate. Written consent was obtained from all participants. Participants were very diverse in terms of racial and ethnic status, and had diverse qualifications. Table 1 gives an overview of the hospices’ representation.

**Table 1:** Research participants

District	Represented hospice #	No of participants from represented hospices	Designation of participants at the represented hospices	Palliative care background of the hospice participants: Medical, psychosocial, spiritual, bereavement
Eden District, Western Cape	Hospice #1	4	Psychosocial Counsellor (1) Social Worker (1) Manager (1) Pastor (1)	Medical (1) Psychosocial (2) Spiritual (1)
	Hospice #2	3	Professional Nurse (1) Chief Executive Officer (1) Psychosocial Manager (1)	Medical (2) Psychosocial (1)
	Hospice #3	1	Chief Executive Officer (1)	Psychosocial (1)
	Hospice #4	1	Nursing Manager (1)	Medical (1)
Cape Town District, Western Cape	Hospice #5	2	Social Worker (1) Medical Doctor (1)	Medical (1) Psychosocial (1)
	Hospice #6	2	Professional Nurse (1) Social Worker (1)	Medical (1) Psychosocial (1)
	Hospice #7	4	Spiritual Care Coordinator (1) Training Facilitator: Professional Nurse (1) Chief Executive Officer (1) Spiritual Care Worker (1)	Medical (1) Psychosocial (1) Spiritual Care (2)
	Hospice #8	1	In-Patient Unit Manager (1)	Medical (1)
	Hospice #9	3	General Manager (1) Patient Care Manager (1) Social Worker (1)	Medical (1) Psychosocial (2)
	Hospice #10	1	Nursing Manager (1)	Medical (1)
	Hospice #11	1	Chief Executive Officer (1)	Medical (1)

We based discussions on 13 open-ended questions (Appendix 1). The FGDs were recorded, transcribed, and coded, and we analysed the data thematically.

## Results

We present the key themes in order of prominence.

### Providing relevant spiritual care services in a religiously, culturally, and racially diverse population

By far the most prominent theme from the FGDs focussed on the barriers hindering the provision of effective and relevant spiritual care services within a highly diverse population.

Participants argued that hospice personnel needed to have a basic working knowledge of the main religions in SA.

On the other hand, the ability to engage with patients about spiritual and existential issues without referencing religion was also mentioned as a crucial skill. Participants emphasised the need to be tolerant, sensitive and non-judgemental.

As a participant from Hospice #2 put it:

*I was sitting here thinking that should the whole spirituality curriculum then not focus a whole lot on those who do the training to reach highest level of self-awareness because when you are self-aware, your ability to tell and convey to others their worth and their value within their existence is just so much easier … because I think that we are kind of stuck in the thing about spirituality equals religion … it doesn’t and to break that mind-set in people, especially in Christians, I find very difficult.*

According to participants, this requires self-knowledge and openness:

*And if it’s about self-awareness, then if you’re not comfortable with homosexuality for example, it would affect you if your patient is requiring, or if your patient who is homosexual needs your help …*

(Participant of Hospice #5)

#### Racial, cultural and linguistic diversity

Participants believed that patients should ideally receive care in their mother tongue. However, the resources required are not often there:

*The other thing of course we started looking at* (was) *for Xhosa speaking patients we want somebody that can support them in their mother tongue language.*

(Participant of Hospice #5)

Specific mention was made of the need to know and respect traditional African beliefs, spiritual practices and bereavement rituals. Hospices need to be tolerant and sensitive to African bereavement rites; and to allow space for these to take place at the hospice in a way that does not negatively impact on the other patients. This is how a participant from Hospice #7 put it:

*… the patient died, and the family came back for the spirit … they want to catch the spirit and they ask us if we can allow them to come catch that spirit because they believe* (after the) *patient* (died) *… the spirit stayed behind … we had to* (allow for this ritual)*, but in the specific way where we say we will give you this part of the area* (hospice) *because we have to think about our other patients and protect them and they don’t see somebody is looking for a spirit here. So we make space for them and they came and catch the spirit and they spent a few hours in ways to get the spirit and then eventually they leave.*

On the other hand, participants noted that patients did not always want a representative from their official religion or speaking their mother-tongue. Some patients may want spiritual guidance and support from a person with whom they have built a close relationship. Navigating the territory between trying to make appropriate spiritual care available on the one hand, and allowing for choices which violate assumptions about cultural and spiritual differences on the other, could be a challenge in practice. As a participant from Hospice #9 put it:

(A Muslim, Afrikaans-speaking patient) *overheard me speaking to other patients after the* (Christian Xhosa-speaking) *pastor had visited … about what is going on in spirituality, in the Christian work.* (The Muslim patient asked me): *“Can the pastor come pray for me?” I said, “We’ve called your Imam* (Islamic priest)*; he’s going to help you.” But she keeps on asking* (for the Christian pastor)*. So there comes a time that they don’t understand their religions, I would rather say that*.

### The organisational context

Participants felt that spiritual care services were an integral part of the palliative care Inter-Disciplinary Team (IDT), whether provided by a designated person or by a team. It was felt that all IDT members should be able to support the patient’s spiritual needs. Hospice participants went on to list intangible resources such as time and effort as a crucial part of spiritual care services.

#### Hospice-specific palliative care team dynamics and organisational culture

Participants affirmed that working in an IDT helped provide a deeper, more holistic understanding of the patient’s situation and challenges. Having a structured inclusion of spiritual care into the IDT consultations and patient assessments was reported to be exceedingly helpful.

On the other hand, participants reported that some colleagues were not comfortable working with spiritual issues. Working in a team would allow such colleagues to hand over to other, better equipped colleagues. As Hospice #1 participant put it:

*So for me it’s working with what you have* (the staff) *and enable that to be completely comfortable and open and that’s where we start, because … a lot of the staff cannot do it* (spiritual care)*. They want to run when there’s an existential need expressed* (by a patient) *…*

There was an expressed need to create spaces where all team members bring different professional, personal and spiritual schools of thought to the table and allow for those differences to be acknowledged, recognised and respected. This creates a tolerant, pluralistic atmosphere for patients as well. Indeed, the smooth functioning of the IDT was viewed as so important that some hospices hired spiritual care staff to fit the team and not necessarily all the ideal requirements of the spiritual care post. In recruitment processes in many hospices, the larger team often gave input into the recruitment and selection of a prospective spiritual care staff member, to ensure that they could all work well together. A participant from Hospice #11 explains:

*We trust each other … when we hire … we hire for a fit on the team … the person that gets chosen may not have all the qualifications but if they fit in the team … we can grow* (that person)*.*

Having a larger team that worked together on the patient’s overall care plan, and particularly the patient’s spiritual care plan, could also ensure that adequate support was provided to the staff, which could reduce stress and burnout. Staff often worked in situations that were difficult or uncomfortable for them. Working in teams could be a better option in difficult situations where workers were struggling, emotionally or professionally, as a participant from Hospice #5 puts it:

*The ability to know even if you are trained, that if you can’t handle a situation, that you need help. I have seen many people go under* (burn out) *because they take on more and more.*

*You don’t go in alone because two is stronger than one.*

The organisational culture of the hospice was cited as another crucial element. Hospice #10 participant explains:

*What culture the organisation lends itself to … whether spirituality is embraced or not … will set the tone in terms of the spiritual care* (that it provides or does not provide)*.*

*… the culture of spirituality … in this organisation … is how we do things and people who come in new will automatically sense the culture of that organisation.*

The calibre of staff working in the spiritual care service team was also mentioned as a crucial factor affecting the quality of spiritual care services. In addition to skills that can be acquired through training and development, staff needed to be empathetic and open to continuous learning, feedback and self-improvement. There was a difference of opinion as to whether there should be dedicated spiritual care service staff or whether all hospice staff should be trained to offer spiritual care services:

*… we have a* (formally employed) *spiritual care worker, volunteer spiritual care worker that sits on our IDT, and are* (present) *at all our meetings and we also have a counsellor and our social worker, nurses, doctors.*

(Representative of Hospice #12)

However,

*Ja* (Yes)*, so for me … my thinking is that in terms of addressing spiritual existential needs, one would have to situate* ***all*** *members of the hospice team that at any given time or any given point in the journey with that patient, they are open and available to address any existential need because you can’t box that need at any point in time.*

(Representative of Hospice #11)

Having dedicated spiritual care staff could allow for more cost-effective training and skills development for fewer people. However, as the patient decides with whom they wish to talk about spiritual and existential issues, having only one or two spiritual care staff could limit the patient’s freedom of choice. Having designated staff also limits the availability of spiritual care services to patients, as patients could decide to talk about these issues whenever the moment seems right to them. The designated spiritual care services staff may not be present at that specific moment, and the present staff would have to deal with the matter as best they can. This could have a negative impact on the quality of spiritual care services provided to the patient.

#### Conflicting demands

The conflicting demands of the patient’s wellbeing – balancing physical care with emotional and spiritual care – contributed additional complexity. All needs were urgent, but finding time to focus on spiritual care in the face of urgent physical care needs could be challenging. There are significantly fewer spiritual care workers compared to other professions represented in hospices.

Participants noted that it is challenging to assess the impact of spiritual care, and this in turn makes it difficult to assess what best practice may be:

*And then I just need to say that most of the time we find out that the value of what we do is only seen after we’ve seen the patient and family and up to two, three months, four months. We get a card or we get a phone call to say thank you that you have helped us through this journey. And every so often our doctor here will come back to me and say* (representative’s name)*, what did you do to that man* (patient)*? And then I will say, well, you asked me to do some support and uh the guy never wanted to be compliant or didn’t want to work along with the doctor and the nurse and was always angry or aggressive maybe. Then after I’ve seen the person* (patient)*, the doctor comes back and wants to know what I did. Well, I think I did what I was asked to do*.

(Hospice #11 representative)

## Discussion

Much of what was discussed by our participants is similar to what has been reported in the literature [24–26], but the local context of huge diversity, great inequality, and scarce resources, places particular demands on local hospices. The responses of our participants enable us to add to the discussions raised by the few studies in the global South [15, 27, 28]. The issue of diversity in spiritual care is now being discussed in diversifying countries like, for example, the USA [29–31] and in other African contexts [32–34]. There are similarities between our findings and those in other diverse contexts; the particular South African history, though, does create a political context which is probably unique [35].

It is also important to note that the psychology of having to deal with very difficult issues – illness and death – may well have influenced our participants’ responses and their discussion of spirituality issues in the South African context. It is well established that people who deal with human pain and suffering may well experience a degree of what has been termed secondary traumatisation or compassion fatigue [36, 37]. The emotional experience of dealing with suffering and death may, of course, also have positive effects and build resilience in carers [38].

It was clear from our data that all our participants indicated that respect for spiritual, cultural and religious diversity is important, and all seemed to draw on their experiences to come to the views they shared with us. It is also important to note that some participants indicated their rejection of a simplistic and neat categorisation, and some will also be troubled by the rather simplistic categorisation of people into separate boxes according to their ascribed cultural and religious status. In SA, given the legacy and continuing pain of its colonial and apartheid past [23], cultural differences map on to concerns about racial oppression and injustice. For South Africans committed to the best possible culturally appropriate spiritual care, it may be more difficult than elsewhere to come to terms with the fact that some people prefer spiritual care from people who are very different culturally and linguistically from them. In this regard, it is interesting that one of our participants, confronted with a situation like this, and wishing to provide the most appropriate care, said, *“So there comes a time that they don’t understand their religions”*. Here, the care receivers are (inadvertently, we think) portrayed as deficient – not understanding their own religions – rather than having, as all people are entitled to have, complex and contradictory positions in relation to cultural and spiritual diversity.

Given that much of what is written about spiritual care is from contexts where the patient and spiritual carer have the same cultural, linguistic and religious background, it is appropriate that the literature does call upon readers to recognise and be sensitive to cultural and religious differences when they do exist. In SA, though, not uniquely, but crucially for our participants, the idea of protecting cultural differences has been used historically to justify segregation and even oppression [21]. There is a paradox in this – as our participants note, culturally appropriate care is important, but rigid ideas about inherent difference may problematically reinforce old stereotypes and not lead to the best care. It is easy to say in the abstract that in spiritual care we wish to follow the lead of care recipients themselves, but in a context in which there is great anxiety about not wishing to impose [23] or to be culturally insensitive, it may be challenging to deal with people whose expressed needs violate common assumptions about what culturally-based needs are. The wish to be culturally sensitive may, paradoxically, make carers in a divided society anxious and hesitant to provide what patients want, for fear of appearing culturally insensitive. This is an issue globally [39], but takes on a particular valence in the South African context. This, we believe, is a crucial issue to address in the development of spiritual care in SA. In this regard, a recent South African article about family care in the context of intellectual disability [40] notes that even when there are obvious cultural and racial differences between carers and those they care for, there may be surprising areas of commonality between them. Good spiritual care recognises and appreciates differences, just as it honours similarities, and, crucially, allows care recipients themselves to position themselves in contradictory and inconsistent ways in relation to these issues. Finding the right way to engage in this context is challenging, and, of course, there is no single and clear right way.

Given resource constraints, a further aspect to these challenges is the reality that at any one time, people with vastly different views and backgrounds will be recipients of care in the same hospice. Our participants recognised that precisely by wanting to embrace the many cultures their patients represent; hospices sometimes inadvertently fail to meet the cultural needs of all their patients. For example, most patients and families that come into hospices come from heterosexual marriages or relationships. Further, patients admitted into hospices are generally older and less likely to be fully exposed to the concepts of same-sex marriages and partnerships, and the many gender related relationship permutations that have recently emerged and are protected under the South African constitution. Some patients may be offended and upset by being in a small and confined hospice together with people in same-sex relationships, for example, but all people are equally entitled to care and to feeling safe. Where resources are few, this equity may be difficult to achieve in practice.

Similarly, allowing patients and their families to engage in cultural practices at the hospice may be a contentious debate. It is easy to declare that all patients should have equal rights to practice their cultures at the hospice and that the hospice has the obligation to offer freedom of expression to the patients with culturally divergent practices. But as our participant mentioned, in the case of *“the patient died, and the family came back for the spirit … they want to catch the spirit …*” , the reality is that most hospices do not have the luxury of space or private patient rooms to assign patients of similar cultures to certain wings of the hospice. The custom of *“catching of the spirit”*, which according to tradition must take place on the ward where the patient died, and is an important spiritual ritual, may upset other patients and families, and in fact they may experience witnessing the ritual as a violation of their own spiritual rights. Navigating such diversity is challenging, at best, and calls for creative and reality-based solutions.

We do not offer any solutions but we offer these challenges as topics for further debate, workshops and focused discussions towards the development of a spiritual care response that embraces the cultural diversity as well as the politics of cultural diversity in SA.

These issues, and all others are profoundly affected by resource constraints. All the hospices that participated in this study are non-governmental organisations (NGOs). NGOs, in a South African context, depend largely on funding from the public to sustain their work. Fundraising is always challenging and is likely to become much more so in the context of the COVID-19 pandemic and its aftermath. Where resources are constrained, there is a danger that there will be a focus, understandably, just on medical care. The gains made by the informality of spiritual care offered in many hospices by unpaid volunteers may be lost if even the very low costs of administering such a service and paying expenses like transport for volunteers may come to be seen as unaffordable.

However, and despite the reality of these challenges, participants felt that the cash versus care argument should not lead to nihilism but to possible rethinking of how care can be best and most efficiently delivered. This represents a real challenge for the future.

## Conclusion

It emerged quite clearly from this study that rendering spiritual care services within a diverse palliative care setting, such as the Western Cape, SA, was both essential and challenging. Hospice staff need to be in possession of a broad range of critical skills, knowledge and expertise in order to provide quality spiritual care services against the backdrop of deeply entrenched external constraints such as racial prejudices, religious difference, and social and cultural segregation. The highly individualised nature of spiritual care services, the diverse population, and individual hospices’ physical and human resource constraints added additional levels of complexity to spiritual care and palliative care. If spiritual care is to have its rightful place in palliative care globally, these are issues which need to be borne in mind.

## Data Availability

The datasets used and/or analysed during the current study are available from the corresponding author on reasonable request.

## Appendix

**Table 2:** Guiding questions for semi-structured focus group discussions

*Positioning spiritual care services as offered by the hospices in the Western Cape*	
1.	What is your understanding of the South African National Policy Framework and Strategy on Palliative Care 2017–2022 (NPFSPC)?
2.	How does the NPFSPC influence service delivery at your hospice?
3.	Does your hospice offer spiritual care services?
4.	Describe the nature of the spiritual care intervention provided by your organisation.
5.	What has the benefit been to the patient/family after receiving spiritual care intervention?
*The role of spiritual care within a hospice in a diverse SA context*	
6.	Describe the role of spiritual care as one of the four pillars of palliative care within your hospice.
7.	How would you describe the role of the spiritual care workers within the Inter-Disciplinary Team (IDT) or Multi-Disciplinary Team (MDT) at your hospice?
8.	Given the range of diversity of SA, how would you say this affects the thinking of spiritual care delivery within your hospice?
*Skills and training needs of a spiritual care worker towards the development of a spiritual care training curriculum*	
9.	What skills are needed to provide spiritual care services?
10.	What resources are needed to provide spiritual care services?
11.	Are your spiritual care workers trained in spiritual care?
12.	What would you consider to be the curriculum needs of spiritual care workers?
13.	What would you consider to be the curriculum content of a spiritual care training programme within a hospice palliative care setting in SA?

## Abbreviations

FGDs: Focus group discussions
IDT: Inter-Disciplinary Team
NGO: Non-governmental organisation
SA: South Africa
